# A Hyperspectral Remote Sensing Image Encryption Algorithm Based on a Novel Two-Dimensional Hyperchaotic Map

**DOI:** 10.3390/e27111117

**Published:** 2025-10-30

**Authors:** Zongyue Bai, Qingzhan Zhao, Wenzhong Tian, Xuewen Wang, Jingyang Li, Yuzhen Wu

**Affiliations:** 1 College of Information Science and Technology, Shihezi University, Shihezi 832002, China; 20232008005@stu.shzu.edu.cn (Z.B.); twz_shzu@163.com (W.T.); wangxuewen@shzu.edu.cn (X.W.); lijing-yang@stu.shzu.edu.cn (J.L.); 2 Geospatial Information Engineering Research Center, Xinjiang Production and Construction Corps, Shihezi 832002, China; 3 Industrial Technology Research Institute, Xinjiang Production and Construction Corps, Shihezi 832002, China; 4 School of Information Network Security, Xinjiang University of Political Science and Law, Tumxuk 843900, China; wuyuzhen@stu.shzu.edu.cn

**Keywords:** hyperchaotic map, hyperspectral image encryption, chaotic performance evaluation, security analysis, DNA encoding

## Abstract

With the rapid advancement of hyperspectral remote sensing technology, the security of hyperspectral images (HSIs) has become a critical concern. However, traditional image encryption methods—designed primarily for grayscale or RGB images—fail to address the high dimensionality, large data volume, and spectral-domain characteristics inherent to HSIs. Existing chaotic encryption schemes often suffer from limited chaotic performance, narrow parameter ranges, and inadequate spectral protection, leaving HSIs vulnerable to spectral feature extraction and statistical attacks. To overcome these limitations, this paper proposes a novel hyperspectral image encryption algorithm based on a newly designed two-dimensional cross-coupled hyperchaotic map (2D-CSCM), which synergistically integrates Cubic, Sinusoidal, and Chebyshev maps. The 2D-CSCM exhibits superior hyperchaotic behavior, including a wider hyperchaotic parameter range, enhanced randomness, and higher complexity, as validated by Lyapunov exponents, sample entropy, and NIST tests. Building on this, a layered encryption framework is introduced: spectral-band scrambling to conceal spectral curves while preserving spatial structure, spatial pixel permutation to disrupt correlation, and a bit-level diffusion mechanism based on dynamic DNA encoding, specifically designed to secure high bit-depth digital number (DN) values (typically >8 bits). Experimental results on multiple HSI datasets demonstrate that the proposed algorithm achieves near-ideal information entropy (up to 15.8107 for 16-bit data), negligible adjacent-pixel correlation (below 0.01), and strong resistance to statistical, cropping, and differential attacks (NPCR ≈ 99.998%, UACI ≈ 33.30%). The algorithm not only ensures comprehensive encryption of both spectral and spatial information but also supports lossless decryption, offering a robust and practical solution for secure storage and transmission of hyperspectral remote sensing imagery.

## 1. Introduction

With the rapid advancement of remote sensing technology, hyperspectral images have found extensive applications in fields such as land mapping, disaster detection, environmental monitoring, agricultural monitoring, and military reconnaissance, owing to their unique advantages of high spatial resolution (sub-meter level), high spectral resolution (less than 10 nm), and high temporal resolution (hourly response) [1,2]. However, hyperspectral remote sensing images contain not only spatial texture features but also spectral information characterized by continuous bands [3]. This type of geospatial data faces significant security threats during storage, transmission, and sharing. If such data is intercepted, critical information could be extracted through spectral feature inversion, posing substantial risks to the security of hyperspectral remote sensing images. Therefore, establishing a robust security protection framework for hyperspectral remote sensing imagery is of great importance. To safeguard image information, researchers have developed various techniques, including image encryption [4,5,6,7] and watermarking [8,9,10,11]. Among these, image encryption stands out as one of the most direct and effective methods for ensuring image security [12]. It transforms a plain image into a highly secure, completely indiscernible cipher image. Only with the correct key can the original image be fully restored.

Chaos theory is one of the most widely applied and effective methods in the field of image cryptography [13,14,15,16]. Chaotic systems are characterized by sensitivity to initial conditions, ergodicity, pseudo-randomness, and unpredictability [17], features that share many common attributes with the principles of image encryption. Chaotic systems can be broadly categorized into continuous-time chaotic systems, typically described by differential equations, and discrete-time chaotic maps, which generate sequences through iterative computations. Discrete chaotic maps, such as Logistic, Tent, Cubic, Chebyshev, and their various coupled or transformed forms, are particularly favored in image encryption due to their computational efficiency and ease of implementation. Compared to one-dimensional (1D) chaotic maps, high-dimensional maps typically possess more complex structures and offer superior chaotic performance. Hyperchaotic systems, a subset of chaotic systems characterized by at least two positive Lyapunov exponents, exhibit more complex dynamical behaviors, higher randomness, and stronger unpredictability, making them highly suitable for high-security image encryption applications.

In recent years, researchers have proposed various novel 2D chaotic maps and applied them to image encryption algorithms. Hua et al. [18] introduced a new 2D Sine-Logistic modulation map (2D-SLMM), derived from the Logistic and Sine maps, and applied it in a chaotic magic transform (CMT) to efficiently alter image pixel positions. Zhu et al. [19] proposed a 2D Logistic-modulated-sine-coupling-logistic chaotic map (2D-LSMCL), which modulates the Sine map using a Logistic map and couples the modulated result with another Sine map. Teng et al. [20] proposed a 2D cross-coupled hyperchaotic map based on Logistic and Sine maps (2D-CLSS). Their results demonstrated that the system exhibits good ergodicity and a wide range of hyperchaotic phenomena, and it was applied to a new IES. Hu et al. [21] proposed a novel cross-coupled 2D chaotic map combining Sine and Logistic systems, which achieves a hyperchaotic state compared to traditional Sine and Logistic systems.

Concurrently, continuous-time chaotic systems, particularly those based on memristors, have also shown significant potential in image encryption. For instance, studies on bursting firings in memristive Hopfield neural networks, novel 4D discrete hyperchaotic maps based on parallel and cascade memristors, and 6D multistable memristive chaotic systems with a wide range of hyperchaotic states have demonstrated complex dynamics suitable for cryptographic applications [22,23,24]. While these continuous systems offer rich dynamical behaviors, their implementation often requires solving differential equations, leading to higher computational complexity compared to discrete maps. This computational overhead becomes a critical concern when encrypting large-volume hyperspectral remote sensing images, which often reach gigabyte (GB) levels. Therefore, this work focuses on designing a discrete hyperchaotic map that balances superior performance with computational efficiency.

The security of these chaos-based image encryption algorithms depends on both the structure of the encryption algorithm and the chaotic performance of the underlying chaotic map. On the one hand, if the encryption structure is inadequately designed, it may be successfully breached by various cryptanalytic attack methods [25]. Furthermore, statistical security evaluations are necessary but not sufficient conditions for proving cryptographic strength. A comprehensive security analysis must also consider resistance against known cryptanalytic techniques. Recent cryptanalysis works have revealed vulnerabilities in several chaos-based image encryption schemes, highlighting the importance of designing algorithms that can withstand chosen-plaintext and known-plaintext attacks [26,27,28]. On the other hand, with the rapid development of chaos analysis techniques, researchers have found that some existing chaotic maps possess security vulnerabilities due to weak chaotic performance [29,30,31], which consequently leads to security issues in the dependent encryption algorithms [32]. Therefore, designing encryption algorithms with higher security strength and developing chaotic systems with better performance are crucial for advancing chaos-based image encryption technology. Accordingly, this paper proposes a new 2D chaotic map named 2D-CSCM. Its chaotic performance will be rigorously tested and analyzed through various methods, including scatter plots, bifurcation diagrams, Lyapunov exponents, and sample entropy.

In 1998, Fridrich J. first proposed a digital image encryption algorithm based on a chaotic system, which divided the encryption process into two stages: scrambling and diffusion [33]. Subsequently, numerous researchers have continued to propose a multitude of image encryption schemes based on chaotic systems. Wang et al. [34] investigated an image encryption algorithm based on multi-objective particle swarm optimization (MOPSO), DNA encoding sequences, and a 1D Logistic map. Liu et al. [35] proposed a Sin-Arcsin-Arnold multi-dynamic random non-adjacent coupled map lattice (SAMCML) model and utilized it to design an encryption scheme for protecting medical multi-images. Through designed 3D-Fisher transformations and DNA operations, the scheme achieves random cross-plane scrambling and efficient diffusion. Wang et al. [36] proposed a color image encryption algorithm based on a two-dimensional (2D) hyperchaotic system, a three-dimensional (3D) L-shaped transformation, and DNA crossover and mutation. This method constructs an image cube and performs scrambling and diffusion operations on it using chaotic sequences. Sharma et al. [37] proposed an adaptive image encryption algorithm based on the Harris Hawk Optimization (HHO). The image is first decomposed into four sub-images of the same dimension via HVD decomposition for block-based pixel disruption. In the diffusion stage, bit-level XOR operations are performed with a random image generated by the Lorenz equations. Although mainstream research, including the aforementioned work, has achieved considerable results, its focus has predominantly been on the encryption of conventional digital images (e.g., RGB images, grayscale images).

Hyperspectral remote sensing images differ significantly from conventional digital images. The underlying data structure of conventional digital images (e.g., RGB or grayscale images) typically consists of a single-layer or three-layer pixel matrix. In contrast, hyperspectral remote sensing images are composed of dozens to hundreds of continuous spectral bands, each containing its corresponding Digital Number (DN) values. These DN values represent not only brightness but also physical properties such as reflectance and temperature of ground objects. Furthermore, the bit depth of DN values in hyperspectral images often exceeds 8 bits (0–255). More critically, hyperspectral images contain continuous spectral-dimensional information [38] absent in conventional images, forming unique spectral curve characteristics. Existing encryption algorithms designed for conventional digital images lack specialized mechanisms to address these hyperspectral data attributes. Consequently, to achieve effective chaotic encryption for hyperspectral remote sensing images, there is a pressing need for an encryption algorithm capable of handling their multi-dimensional data structure, high bit-depth DN values, and spectral domain features. Moreover, the single-scene data volume of hyperspectral imagery is enormous, often reaching gigabyte (GB) levels, which imposes higher demands on the security of the encryption algorithm, necessitating chaotic systems with superior performance. To address the challenges outlined above, this paper proposes a hyperspectral remote sensing image encryption algorithm based on the 2D-CSCM hyperchaotic map. Targeting the underlying data structure and spectral domain characteristics of hyperspectral data, the algorithm employs a layered processing strategy—comprising spectral dimension encryption, spatial pixel scrambling, and bit-level diffusion based on DNA encoding rules—to protect the spectral curve profiles and achieve comprehensive encryption of the entire image’s information.

In summary, the main contributions of this study are as follows:

(1) A novel 2D hyperchaotic map (2D-CSCM) is proposed, integrating Cubic, Sinusoidal, and Chebyshev maps. It demonstrates superior hyperchaotic behavior, including a wider hyperchaotic parameter range and enhanced randomness, as validated by Lyapunov exponents, sample entropy, and NIST tests.
(2) A dedicated spectral domain encryption method is designed, effectively concealing critical spectral curve features while preserving spatial readability—a crucial requirement for hyperspectral image processing.
(3) A comprehensive encryption framework is developed, incorporating the proposed hyperchaotic map, spectral scrambling, spatial permutation, and a bit-level DNA diffusion mechanism tailored for high bit-depth DN values, ensuring robust security for hyperspectral imagery.

The remainder of this paper is organized as follows. Section 2 introduces the 2D-CSCM chaotic map and its chaotic performance. Section 3 describes the framework and details of the proposed hyperspectral remote sensing image encryption method based on 2D-CSCM. Section 4 provides a comprehensive security analysis of the proposed encryption algorithm. Finally, Section 5 and Section 6 present the discussion and conclusions.

## 2. 2D-CSCM Hyperchaotic Map

Chaotic maps are commonly employed in cryptography to generate pseudo-random sequences, forming the foundation of chaos-based image encryption methods. Therefore, this section will first introduce the proposed 2D-CSCM (2D Cubic–Sinusoidal–Chebyshev Map) hyperchaotic map. Its performance will then be evaluated through a series of metrics to demonstrate superior chaotic behavior, justifying its application in the encryption algorithm proposed in this paper.

### 2.1. Definition of the 2D-CSCM Hyperchaotic Map

This paper proposes a two-dimensional hyperchaotic map named 2D-CSCM, which integrates the cubic map, sinusoidal map, and Chebyshev map. The map accepts two input states, $x_{n}$ and $y_{n}$, and produces two output states, $x_{n+1}$ and $y_{n+1}$. The mathematical formulation of this cross-coupled map is given by Equation (1), where $f_{1}$ and $f_{2}$ are typically chaotic maps, and the output states are derived from computations using these functions applied to the input states:

(1) $$\left\{\begin{array}{c} x_{n+1}=f_{1}\left(x_{n},y_{n}\right)\\ y_{n+1}=f_{2}\left(x_{n},y_{n}\right) \end{array}\right.$$

In this work, we enhance the cross-coupled structure by incorporating the cubic map, sinusoidal map, and Chebyshev map, along with trigonometric sine and cosine functions, to propose the 2D-CSCM hyperchaotic map. The specific mathematical expression is provided in Equation (2):

(2) $$\left\{\begin{array}{c} x_{n+1}=\sin(\pi (2.595x_{n}\left(1-{x_{n}}^{2}\right)+\mu {y_{n}}^{2}\sin\left(\pi y_{n}\right)+\cos(4\pi \arccos(y_{n})))\\ y_{n+1}=\cos(\pi (\mu y_{n}\left(1-{y_{n}}^{2}\right)+2.3{x_{n}}^{2}\sin\left(\pi x_{n}\right)+\cos(4\pi \arccos(x_{n}))) \end{array}\right.$$

where μ denotes the control parameter, and $x_{n+1}$, $y_{n+1}$ represent the subsequent states of the current inputs $x_{n}$ and $y_{n}$, respectively.

The rationale for selecting this specific combination of maps lies in their complementary chaotic properties. The cubic map serves as the core nonlinear component providing robust chaotic dynamics. The sinusoidal map introduces a periodic element that, when nonlinearly coupled, helps in disrupting periodic patterns and enhances sensitivity. The Chebyshev map contributes its distinct orthogonal and ergodic characteristics, which diversify the algebraic structure of the system and aid in achieving a more uniform distribution of output sequences. This synergistic design aims to create a hyperchaotic map with superior performance.

Furthermore, the constants 2.595 and 2.3 in Equation (2) were determined through a numerical optimization process. We performed a parameter sweep, evaluating the chaotic strength using the Lyapunov exponent as the key metric. These specific values were identified as optimal for maximizing the Lyapunov exponents and ensuring a wide hyperchaotic parameter range for μ, thereby guaranteeing the map operates at its peak chaotic performance for cryptographic applications.

The map exhibits chaotic behavior over a broad parameter range $\mu \in \left[0,+\infty )\right.$. The use of sine and cosine functions ensures that the map states remain bounded within $x_{n},y_{n}\in [-1,1]$.

### 2.2. Performance Evaluation of the 2D-CSCM Hyperchaotic Map

The comprehensive comparative analysis presented in this section aims to transcend mere performance benchmarking and delve into the mechanistic advantages of the proposed 2D-CSCM. While many recent 2D chaotic maps, such as 2D-SLMM [18] and 2D-CLSS [21], are constructed by coupling variants of the Logistic and Sine maps, our 2D-CSCM introduces a fundamental architectural innovation by cross-integrating three distinct families of maps: the Cubic, Sinusoidal, and Chebyshev. This synergistic design is hypothesized to be the root cause of its superior dynamics. The Cubic map provides a stronger nonlinear driving force compared to the Logistic map. The incorporation of the Chebyshev polynomial, with its inherent orthogonality and ergodicity, diversifies the algebraic structure and enhances output uniformity. Finally, the nested trigonometric coupling amplifies sensitivity and disrupts periodic patterns. The following quantitative comparisons serve to validate that this structural novelty directly translates into observable performance advantages.

To validate the chaotic effectiveness of the proposed 2D-CSCM, this section conducts a comprehensive evaluation using a series of standard chaotic metrics, including: attractor phase diagrams, bifurcation diagrams, Lyapunov exponents, value distribution histograms, sensitivity to initial conditions, sample entropy, 0–1 test, and the NIST SP 800-22 statistical test suite. For objective comparison, the performance of 2D-CSCM will be benchmarked against several classical and recently proposed chaotic maps, namely the Henon map, 2D-SLMM [18], 2D-LSMCL [19], and 2D-CLSS [21], in order to highlight its advantages in terms of chaotic complexity, randomness, and stability (reliability).

This section presents a comprehensive evaluation of the 2D-CSCM map’s chaotic performance through a series of standard metrics. A key distinction of a hyperchaotic map, as proposed, is the presence of at least two positive Lyapunov exponents. This property, coupled with complex dynamical behaviors like a wide chaotic parameter range and high entropy, makes hyperchaotic maps particularly suitable for high-security cryptography. The following analyses not only validate the hyperchaotic nature of 2D-CSCM but also benchmark its performance against several classical and recent 2D chaotic maps through direct, fair comparisons under identical conditions. The specific parameter settings used for the comparative maps in the following figures are provided in Appendix A for completeness.

#### 2.2.1. Phase Diagram

The trajectory of a chaotic attractor directly reflects the nonlinearity and complexity of the system [39]. For periodic behavior, the trajectory typically appears as a fixed or repetitive curve. In contrast, a bounded chaotic system exhibits trajectories confined to a certain range, yet never repeating or converging. Ideally, a chaotic trajectory should show no fixed pattern and be uniformly distributed throughout the phase space, indicating high randomness in the generated sequences. A uniform phase space distribution suggests that the chaotic map can output well-randomized values.

**Figure 1:**
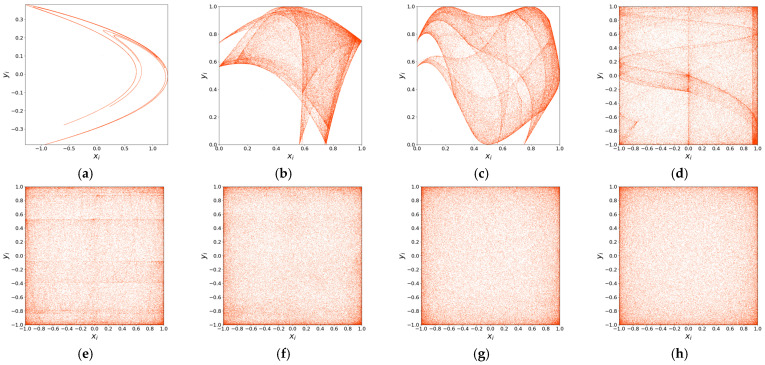
Attractor Phase Diagram. (**a**) Attractor Phase Diagram of the Henon Chaotic Map; (**b**) Attractor Phase Diagram of the 2D-SLMM; (**c**) Attractor Phase Diagram of the 2D-LSMCL; (**d**) Attractor Phase Diagram of the 2D-CLSS; (**e**–**h**) Attractor phase diagrams of 2D-CSCM at μ = 0, μ = 0.1, μ = 2.5, and μ = 200, respectively.

Figure 1 illustrates the attractor phase diagrams of different chaotic maps. Figure 1a–d show the attractor distributions of the compared chaotic maps, while Figure 1e–h present the attractor distributions of the proposed 2D-CSCM with initial values $\left(x_{0},y_{0}\right)=(0.4,0.6)$ under μ=0, μ=0.1, μ=2.5, and μ=200, respectively. The attractor phase diagrams of the comparison maps exhibit clearly fixed trajectories and uneven distributions. In contrast, the trajectory of the 2D-CSCM attractor shows no obvious pattern under chaotic states, with a more uniform distribution overall. Moreover, as the parameter μ increases, the distribution becomes even more homogeneous. These results demonstrate that the 2D-CSCM possesses more complex dynamic behavior and enhanced chaotic properties.

#### 2.2.2. Bifurcation Diagram

**Figure 2:**
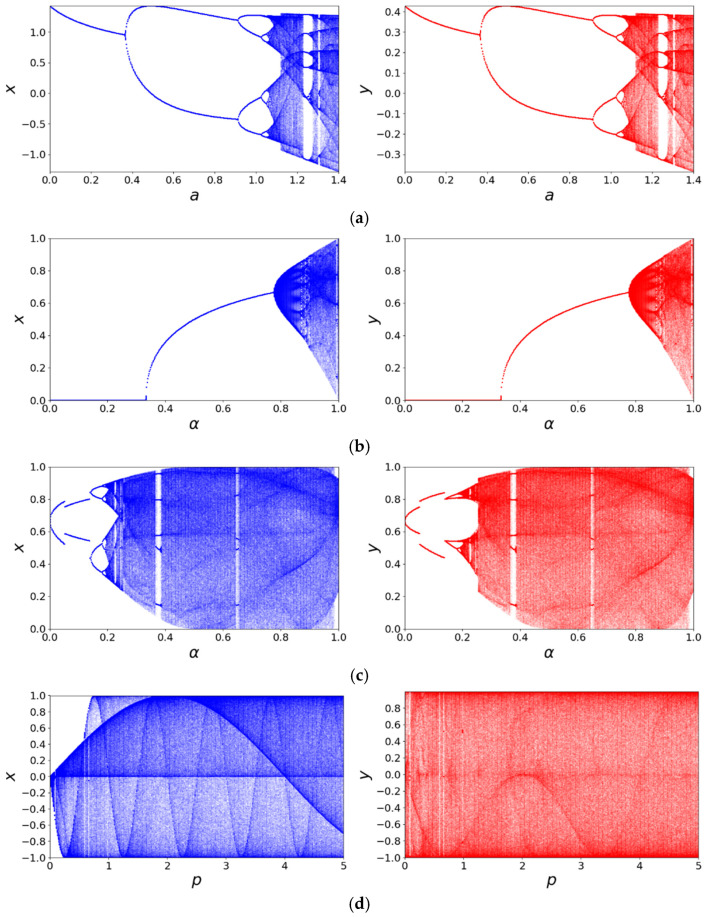

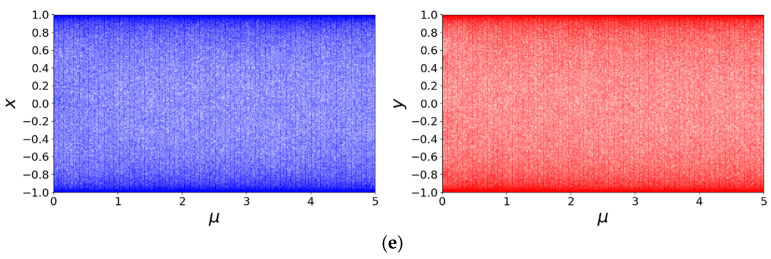
Bifurcation diagram comparison. (**a**) Bifurcation diagram of the Henon Chaotic Map; (**b**) Bifurcation diagram of the 2D-SLMM; (**c**) Bifurcation diagram of the 2D-LSMCL; (**d**) Bifurcation diagram of the 2D-CLSS; (**e**) Bifurcation diagram of 2D-CSCM.

The bifurcation diagram reveals how the qualitative behavior of a chaotic map evolves with a control parameter. By discarding initial transients and plotting the long-term states, it provides a powerful and intuitive visualization of global dynamics, including period-doubling routes to chaos and the existence of periodic windows [40]. Figure 2 shows the bifurcation diagrams of the 2D-CSCM and several comparative chaotic maps. The diagrams reveal that the other maps exhibit chaotic behavior only within limited parameter intervals, and their state values do not fully cover the entire value range. In contrast, the 2D-CSCM maintains chaotic behavior throughout μ∈[0,5], with values entirely distributed within the interval [−1, 1]. The bifurcation diagram of 2D-CSCM demonstrates a more uniform and continuous distribution, indicating higher dynamic complexity and stronger chaotic characteristics. Moreover, 2D-CSCM remains chaotic over an even broader parameter range μ∈[0,+∞).

#### 2.2.3. Lyapunov Exponent

The Lyapunov exponent is a powerful measure for quantifying the degree of chaos in a dynamical system, evaluating the unpredictability and complexity of chaotic sequences it generates [41]. It measures the rate of divergence of nearby trajectories, reflecting the system’s sensitivity to initial conditions. A positive Lyapunov exponent indicates chaotic behavior, high sensitivity to minute changes in initial conditions, and thus unpredictability. In contrast, a negative Lyapunov exponent suggests convergence toward a fixed point or limit cycle, implying stability and predictability. A system with multiple positive Lyapunov exponents is considered hyperchaotic. Hyperchaotic maps generally exhibit more complex and richer dynamic behavior than standard chaotic maps, making them highly suitable for image encryption, as they produce intensely scrambled outputs that are difficult to predict or decrypt without the correct key and algorithm.

**Figure 3:**
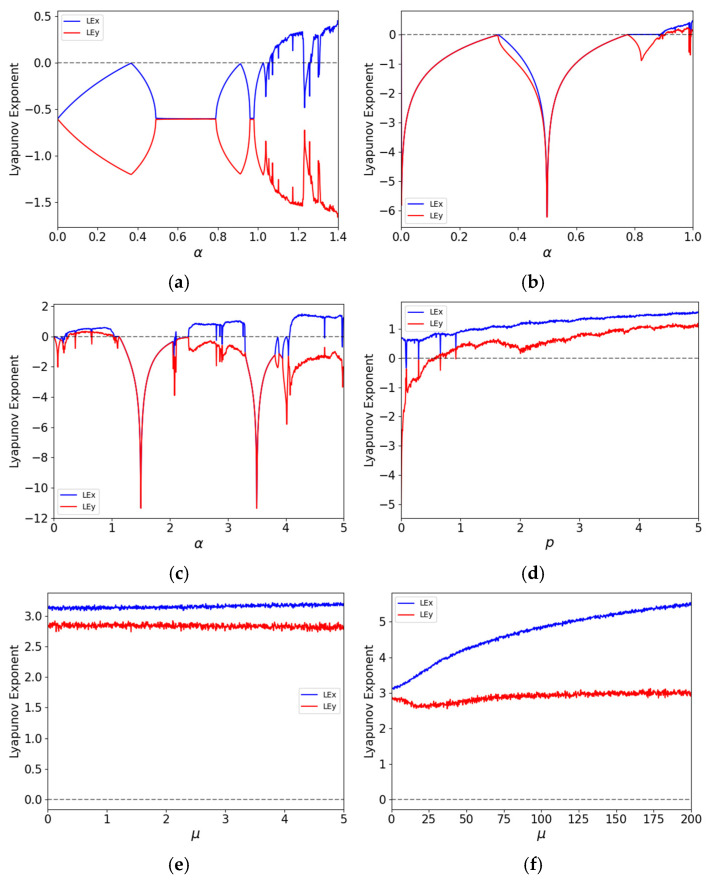
Distribution of Lyapunov exponents. (**a**) Distribution of Lyapunov exponents of the Henon Chaotic Map; (**b**) Distribution of Lyapunov exponents of the 2D-SLMM; (**c**) Distribution of Lyapunov exponents of the 2D-LSMCL; (**d**) Distribution of Lyapunov exponents of the 2D-CLSS; (**e**,**f**) Distributions of Lyapunov exponents of 2D-CSCM under μ∈[0,5] and μ∈[0,200], respectively.

**Figure 4:**
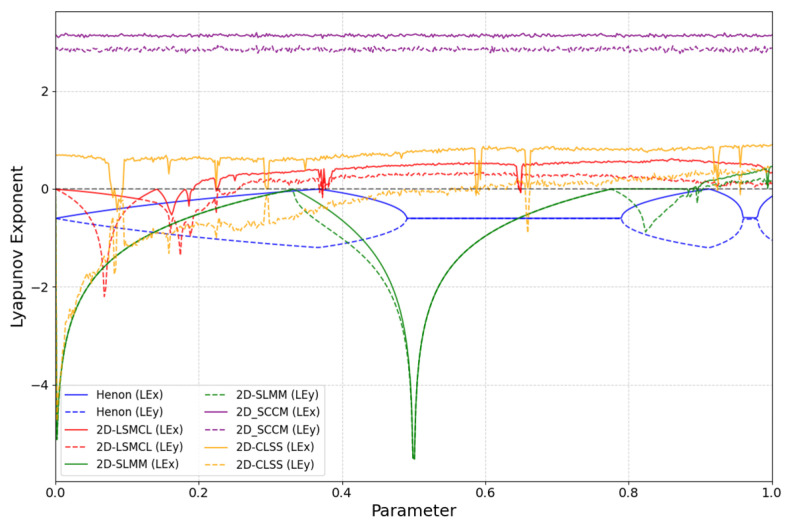
Comparison of Lyapunov exponent curves (Parameter ranging from 0 to 1).

The presence of multiple positive Lyapunov exponents is a definitive marker of hyperchaos. From a cryptographic standpoint, such systems offer superior properties. First, multiple positive LEs indicate exponential divergence in more than one direction within the phase space [42], leading to more rapid and complex mixing of state variables. This directly translates to enhanced sensitivity and higher unpredictability in the generated sequences—a crucial property for confounding statistical analysis. Furthermore, the magnitude of the Lyapunov exponents is of critical importance. A larger positive LE signifies a faster average rate of divergence [43], meaning the system loses correlation with its initial state more rapidly, thereby increasing its short-term unpredictability and resistance to prediction attacks. As evidenced by our results (Figure 3 and Figure 4), the 2D-CSCM not only possesses two positive LEs across a wide parameter range but also maintains large values for these exponents, confirming its superior hyperchaotic strength for cryptographic applications.

In this study, the Lyapunov exponents are computed using the QR decomposition method [44]. The distributions of Lyapunov exponents for 2D-CSCM and other comparative chaotic maps are shown in Figure 3, where Figure 3e,f represent the performance of 2D-CSCM over μ∈[0,5] and μ∈[0,200], respectively. The results demonstrate that 2D-CSCM has two positive Lyapunov exponents across both intervals, proving that it exhibits not only chaotic but also hyperchaotic behavior. In contrast, the other chaotic maps show chaotic characteristics only within limited portions of their parameter intervals. Compared to these maps, 2D-CSCM exhibits larger Lyapunov exponents that remain positive throughout the parameter range, attesting to its stronger chaotic nature. Figure 4 provides a detailed comparison of Lyapunov exponent curves over the interval ∈[0,1]. Moreover, 2D-CSCM maintains its hyperchaotic behavior over the entire range μ∈[0,+∞).

#### 2.2.4. Sensitivity to Initial Conditions

**Figure 5:**
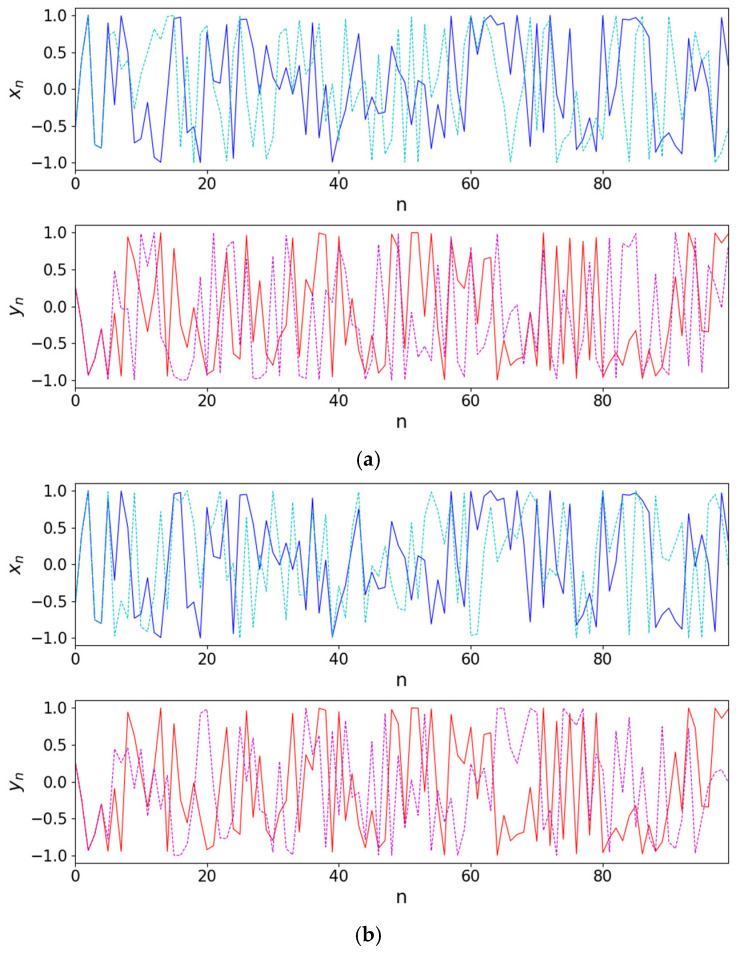
Sensitive dependence on initial conditions is illustrated in the control and parallel runs with initial conditions (**a**) $x_{0}=0.5$, $y_{0}=0.5$(solid line) and $x_{0}=0.5$, $y_{0}=0.5-10^{-15}$ (dashed line); (**b**) $x_{0}=0.5$, $y_{0}=0.5$(solid line) and $x_{0}=0.5+10^{-15}$, $y_{0}=0.5$ (dashed line).

This section focuses on evaluating the sensitivity of the 2D-CSCM to initial conditions. By introducing a minute perturbation to the initial values and comparing the chaotic trajectories of the original and perturbed systems over the same time range, the dependence of the system on initial conditions can be visually assessed [45]. The initial state of the system is set as $x_{0}=0.5$, $y_{0}=0.5$ and a perturbation of magnitude $10^{-15}$ is applied to $x_{0}$ and $y_{0}$, respectively. Figure 5a shows a comparison between the original trajectory ($x_{0}=0.5$, $y_{0}=0.5$) and the trajectory after perturbing $y_{0}$ ($x_{0}=0.5$, $y_{0}=0.5-10^{-15}$); Figure 5b displays the original trajectory and the trajectory after perturbing $x_{0}$ ($x_{0}=0.5+10^{-15}$, $y_{0}=0.5$). It is clearly observed that even under extremely small initial disturbances, the system trajectories diverge significantly, demonstrating the high sensitivity of 2D-CSCM to initial conditions.

#### 2.2.5. Sample Entropy

Sample entropy [46] is used to quantify the oscillation patterns in a time series and is proportional to the complexity of the sequence. It can be calculated using Equation (3):

(3) $$SE\left(m,r,N\right)=-log\frac{S}{P}$$

where m is the embedding dimension, r is the tolerance value, and N is the length of the time series. S and P denote the number of template vector matches using the Chebyshev distance between i and j. For two-dimensional chaotic sequences, the sample entropy typically falls between 0 and 2. A higher sample entropy value generally indicates stronger nonlinearity and complexity in the sequence.

**Figure 6:**
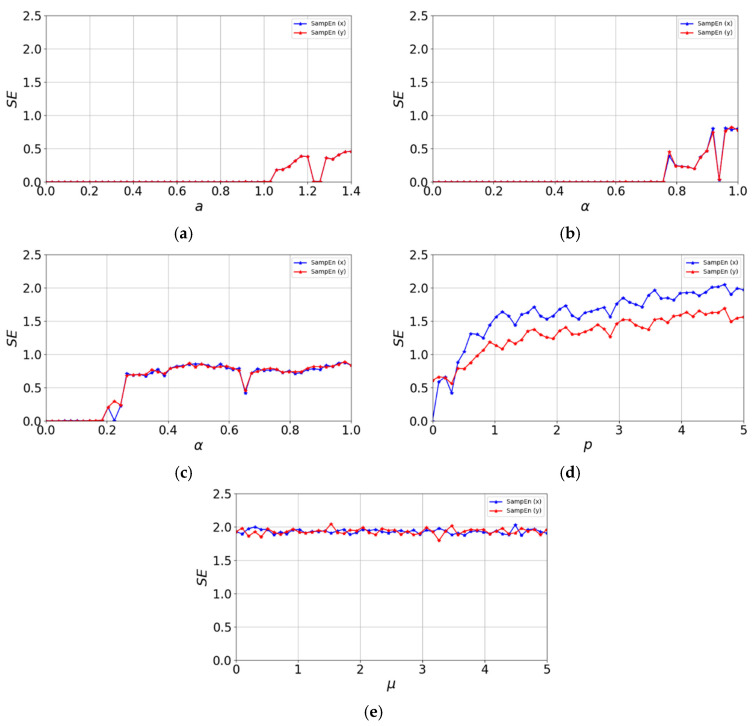
Sample entropy distribution. (**a**) Sample entropy distribution of the Henon Chaotic Map; (**b**) Sample entropy distribution of the 2D-SLMM; (**c**) Sample entropy distribution of the 2D-LSMCL; (**d**) Sample entropy distribution of the 2D-CLSS; (**e**) Sample entropy distribution of 2D-CSCM.

Figure 6 shows the sample entropy performance of the 2D-CSCM and other comparative chaotic maps. The other maps struggle to achieve the ideal value of 2 within their chaotic parameter ranges. In contrast, the sample entropy of 2D-CSCM remains close to 2 across different values of the control parameter μ. Compared to the other maps, 2D-CSCM exhibits sample entropy values much closer to the ideal value throughout the parameter range, indicating superior sequence complexity.

#### 2.2.6. 0–1 Test

The 0–1 test is an important method for evaluating the expansion behavior of chaotic map trajectories, used to identify chaotic dynamics in a time series [47]. For a given time series φ(j), j=1,2,…,N, the test is conducted as follows:

(4) $$p\left(n\right)=\sum_{j=1}^{n}\varphi \left(j\right)\cos\left(\theta \left(j\right)\right)$$

(5) $$q\left(n\right)=\sum_{j=1}^{n}\varphi \left(j\right)\sin\left(\theta \left(j\right)\right)$$

(6) $$\theta \left(j\right)=jc+\sum_{i=1}^{j}\varphi \left(i\right)$$

where c∈(0,2π) is a random constant, and n=1,2,…,N, j=1,2,…,n. The mean square displacement $M\left(n\right)$ is computed as:

(7) $$M\left(n\right)=M_{c}-{\left(E\left(\phi \right)\right)}^{2}\left(\frac{1-cosnc}{1-cosc}\right)$$

(8) $$M_{c}\left(n\right)=\underset{N\to \infty }{\lim}\left[{\left(p\left(j+n\right)-p\left(j\right)\right)}^{2}-{\left(q\left(j+n\right)-q(j)\right)}^{2}\right]$$

(9) $$E\left(\phi \right)=\underset{N\to \infty }{\lim}\frac{1}{N}\sum_{j=1}^{N}\varphi \left(j\right)$$

If $p\left(n\right)$ and $q\left(n\right)$ exhibit Brownian motion, $M\left(n\right)$ grows linearly with time; if the trajectory is bounded, so is $M\left(n\right)$. The asymptotic growth rate $K_{c}$ is defined as:

(10) $$K_{c}=\underset{N\to \infty }{\lim}\frac{lgM\left(n\right)}{lgn}$$

A value of $K_{c}$ close to 1 indicates chaotic behavior in the time series.

**Figure 7:**
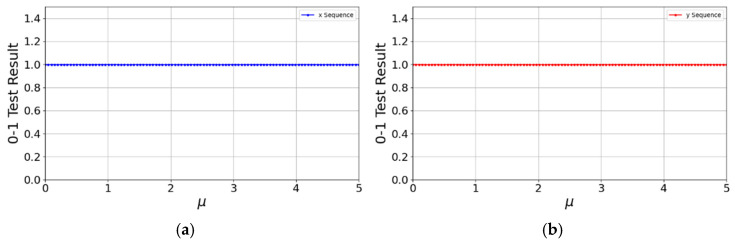
0–1 Test. (**a**) Performance of x sequence in 0–1 test; (**b**) Performance of y sequence in 0–1 test.

We performed the 0–1 test for the control parameter μ ranging from 0 to 5, uniformly sampled at 100 points. As shown in Figure 7, the $K_{c}$ values of the sequences generated by the 2D-CSCM in both the x and y directions are all close to 1. Specifically, the mean $K_{c}$ values for the x and y sequences reach 0.9987 and 0.9991, with variances of 0.0021 and 0.0015, respectively. Since displaying all 100 data points is impractical, we provide these statistical measures which robustly confirm the chaotic nature of the map’s output.

#### 2.2.7. NIST SP 800-22 Test

The NIST SP 800-22 test is a comprehensive statistical suite provided by the National Institute of Standards and Technology (NIST) for evaluating the randomness and statistical properties of binary sequences [48]. It consists of 15 distinct tests. The *p*-value is a statistical measure indicating the probability of observing the data under the null hypothesis. A sequence is considered random for a given test if the *p*-value exceeds the threshold α = 0.01. Table 1 presents the NIST test results for the output sequences of the 2D-CSCM. As shown, the binary streams generated by 2D-CSCM passed all subtests. These results demonstrate that 2D-CSCM can produce long, aperiodic chaotic sequences suitable for image encryption.

## 3. Hyperspectral Remote Sensing Image Encryption Algorithm Based on the 2D-CSCM Hyperchaotic Map

**Figure 8:**
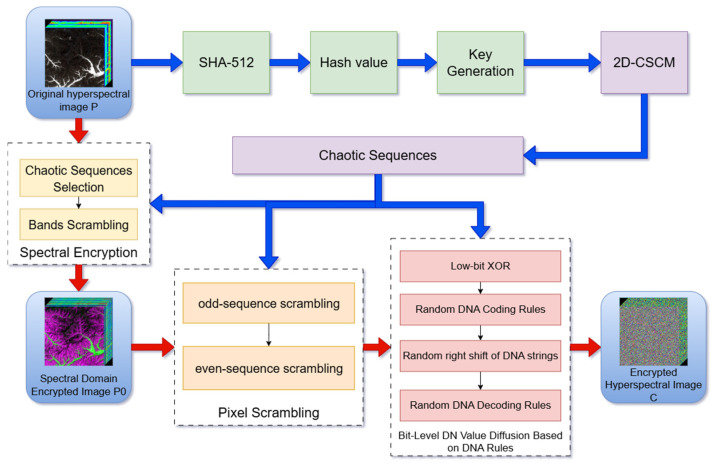
Encryption Algorithm Process.

This section introduces a novel encryption algorithm for hyperspectral remote sensing images, founded on the 2D-CSCM hyperchaotic map. The algorithm enables spectral-domain encryption, full-image spatial encryption, and lossless decryption. The process consists of four key stages: key generation, spectral dimension encryption, pixel scrambling, and DN value diffusion. The flowchart of the proposed encryption algorithm is shown in Figure 8.

The proposed algorithm is a symmetric encryption scheme. For each hyperspectral image to be encrypted, the entire process, including the key generation and the iteration of the 2D-CSCM, is executed once. The initial conditions and parameters (kx, ky, ku) for the 2D-CSCM are derived from the plaintext image via the SHA-512 hash function as described in Section 3.1. These values (kx, ky, ku) constitute the secret key.

This secret key must be securely transmitted to the authorized receiver through a separate channel (e.g., encrypted using the receiver’s public key via an algorithm like RSA). Upon receiving the key, the decryption process is initiated.

The 2D-CSCM is a deterministic mathematical function. Given the same initial key (kx,ky,ku), it will generate an identical sequence of chaotic values regardless of the hardware or software platform (e.g., Intel x86-64, ARM-based systems, Windows, and Linux), provided that the floating-point arithmetic conforms to the IEEE 754 standard. This determinism is a fundamental property of chaotic maps and ensures lossless decryption. Our experiments conducted on different computing environments have confirmed this reproducibility.

### 3.1. Key Generation

As a symmetric image encryption algorithm, the same key must be used for both encryption and decryption. This key is transmitted to the receiver via a network or other communication method. In this algorithm, the encryption key is generated using the SHA-512 hash function. The specific steps are as follows:

Step 1: Input the original image into the SHA-512 hash function to obtain a 512-bit hash value H. This hash value is inherently a binary sequence but is typically represented as a 128-character hexadecimal string for readability. For subsequent computations, we treat H directly as a binary sequence B of length 512.

Step 2: Divide sequence B into 8 subsequences denoted as $K_{0}$ to $K_{7}$, each 64 bits long.

Step 3: Convert $K_{0}-K_{5}$ into 64-bit floating-point numbers, and $K_{6}-K_{7}$ into 64-bit integers.

Step 4: Extract the fractional parts of $K_{0}-K_{5}$ to obtain $K_{fr0}-K_{fr5}$. Then, compute the initial state parameters kx and ky using Equations (11) and (12):

(11) $$kx={(K}_{fr0}+K_{fr1}+K_{fr2})/3$$

(12) $$ky={(K}_{fr3}+K_{fr4}+K_{fr5})/3$$

Step 5: Calculate the intermediate value ku′ using Equation (13), convert it into a 64-bit floating-point number, and take its absolute value to obtain the final parameter ku:

(13) $${ku}^{\prime }=K_{6}⨁K_{7}$$

As a result, ku is a positive number, while kx and ky fall within the range [−1,1]. The values ku, kx, and ky are used as the control parameter and initial states of the 2D-CSCM, respectively, to generate the chaotic sequences required in the subsequent encryption process.

The key generation mechanism is plaintext-dependent, as the hash is derived from the original image. This design provides a desirable property of being resistant to known-plaintext attacks for a single image, as each image has a unique key. However, we acknowledge that under a chosen-plaintext attack (CPA) model, where an adversary can obtain the ciphertext for a chosen plaintext, this dependency could be exploited to analyze the relationship between the plaintext and the key. Nevertheless, for the specific application scenario of securing hyperspectral remote sensing images—which are typically large, unique datasets not subject to arbitrary manipulation by an adversary—the practical risk of a successful CPA is considered low. The primary security guarantees rely on the robustness of the SHA-512 hash function and the hyperchaotic map.

The SHA-512 hash function was selected over SHA-256 for two primary reasons: (1) It produces a longer 512-bit digest, which facilitates the derivation of a more complex and higher-entropy set of initial conditions and parameters (kx, ky, ku) for the 2D-CSCM map, thereby strengthening the key space. (2) SHA-512 is optimized for 64-bit processors, and given the large data volume of hyperspectral images, its computational efficiency on modern computing platforms is comparable to, if not better than, SHA-256 for such sizable inputs.

### 3.2. Spectral Encryption Process

Hyperspectral remote sensing images cover a broad spectral range with high resolution, and their spectral domain often contains crucial information, particularly the spectral curve shapes that reflect material properties. Therefore, encrypting the spectral domain is essential for protecting hyperspectral image data. This subsection describes the spectral encryption process based on the 2D-CSCM. The specific steps are as follows:

Step 1: For a hyperspectral image P of size M×N×K, generate two chaotic sequences X and Y of length K using the 2D-CSCM, where M is the number of rows, *N* the number of columns, and K the number of spectral bands.

Step 2: Determine whether M is even or odd. If M is even, select sequence X as the encryption sequence; otherwise, select sequence Y.

Step 3: Sort the values of the selected encryption sequence in ascending order and generate the corresponding index sequence.

Step 4: Use this index sequence to permute all bands of the image, resulting in the spectrally encrypted image P0.

At this stage, the spectral encryption of the hyperspectral image is complete. This process operates solely on the spectral dimension without altering spatial information, thereby encrypting spectral features while fully preserving the spatial texture and structural content of the image.

### 3.3. Pixel Scrambling Process

The spatial information of a hyperspectral remote sensing image is represented by a pixel matrix. To enhance security in the spatial domain, pixel positions are scrambled. This section describes the pixel scrambling process based on the 2D-CSCM. The specific steps are as follows:

Step 1: For the spectrally encrypted hyperspectral image P0 of size M×N×K, generate two chaotic sequences X and Y of length M×N using the 2D-CSCM for subsequent scrambling;

Step 2: Read all pixels of P0 in a snake-like pattern and flatten them into a one-dimensional pixel sequence ${P0}^{\prime }$;

Step 3: Sort the sequences X and Y in ascending order to generate index sequences $X_{1}$ and $Y_{1}$, where $X_{1}$ will be used to scramble the odd-indexed group, and $Y_{1}$ the even-indexed group;

Step 4: Divide the one-dimensional sequence ${P0}^{\prime }$ into an odd group ${{P0}^{\prime }}_{odd}$ and an even group ${{P0}^{\prime }}_{even}$ based on pixel order;

Step 5: Use X_1_ to reorder the odd group, obtaining the scrambled odd group ${{P1}^{\prime }}_{odd}$; similarly, use $Y_{1}$ to scramble the even group, yielding ${P1\prime }_{even}$;

Step 6: Concatenate ${P1\prime }_{even}$ after ${P1\prime }_{odd}$ to form a fully scrambled pixel sequence P1′;

Step 7: Repeat Steps 5–6 for all spectral bands to accomplish pixel scrambling across the entire dataset;

Step 8: Reshape P1′ back into an M×N×K three-dimensional matrix, resulting in the pixel-scrambled image P1.

### 3.4. Bit-Level DN Value Diffusion Process Based on DNA Rules

Unlike conventional grayscale images, the pixel values in hyperspectral remote sensing images are represented as Digital Numbers (DNs), which may indicate brightness, reflectance, radiance, or other physical quantities, with possible bit depths of 8, 16, or 32 bits. To accommodate the encryption of high bit-depth DN values, this paper proposes a bit-level diffusion method based on DNA encoding rules. The specific steps are as follows:

Step 1: For the pixel-scrambled image P1 of size M×N×K, generate two chaotic sequences X and Y of length M×N×K using the 2D-CSCM for subsequent diffusion;

Step 2: Read the DN values of P1 and convert them into an unsigned integer sequence P1″ with the original bit depth;

Step 3: Convert the floating-point values of chaotic sequence X into binary form, truncate the lower bits corresponding to the DN bit-length, and perform a bitwise XOR operation with P1″ to obtain the binary sequence ${P2\prime }_{bin}$;

Step 4: Compute the DNA encoding rule sequence $X_{e}$ from chaotic sequence X using Equation (14), which determines the DNA encoding mode for each value in ${{P2}^{\prime }}_{bin}$, yielding the DNA-encoded string sequence ${{P2}^{\prime }}_{DNA}$:

(14) $$X_{e}=\left(x_{i}\times 10^{4}\right)mod8$$

The result of $X_{e}$ (an integer between 0 and 7) dynamically selects one of the 8 possible DNA encoding rules for each pixel or data block. The standard set of DNA coding rules employed in this study is defined in Table A1 in Appendix A. For example, if $X_{e}=0$, rule ‘A’ might be used where 00 encodes to A, 11 to T, 10 to C, and 01 to G. Our algorithm does not rely on a single fixed rule but pseudo-randomly selects from all 8 rules based on the chaotic sequence, significantly enhancing the diffusion effect and resistance to analysis.

Step 5: Reverse each DNA string in ${{P2}^{\prime }}_{DNA}$ to obtain the reversed sequence ${{P2}^{\prime \prime }}_{DNA}$;

Step 6: Perform a right circular shift on each string in ${{P2}^{\prime \prime }}_{DNA}$. The shift amount is calculated using Equation (15):

(15) $${num}_{move}=\left(\frac{x+y}{2}\times 10^{8}\right)mod\left(len\left({{P2}^{\prime \prime }}_{{DNA}_{i}}\right)\right)$$

where i denotes the current index;

Step 7: Compute the DNA decoding rule sequence $Y_{e}$ from chaotic sequence Y using Equation (16), and decode the shifted DNA strings to obtain the diffused binary sequence ${P2}^{\prime }$:

(16) $$Y_{e}=\left(y_{i}\times 10^{4}\right)mod8$$

Step 8: Convert the binary values in ${P2}^{\prime }$ back into unsigned integers with the original DN bit depth, resulting in the final encrypted image C.

While DNA-based operations provide a novel and complex layer for diffusion, it is recognized that certain implementations can be vulnerable to specific attacks if the encoding/decoding rules are static or poorly chosen. The primary vulnerabilities often lie in the predictability of the rules. Our scheme mitigates this by (1) dynamically selecting the encoding and decoding rules using the hyperchaotic sequences $X_{e}$ and $Y_{e}$, making them plaintext- and key-dependent, and (2) incorporating non-linear operations like bit reversal and circular shifting within the DNA domain. This dynamic and integrated approach strengthens the algorithm against cryptanalysis that targets static DNA coding rules.

This completes the overall encryption of the hyperspectral remote sensing image.

### 3.5. Decryption Process

As a symmetric encryption scheme, the decryption process is the inverse of the encryption procedure. The deterministic nature of the 2D-CSCM ensures that, given the same secret key (kx, ky, ku), identical chaotic sequences are generated for decryption, regardless of computing platform, provided IEEE 754 floating-point standard is followed. This determinism guarantees lossless decryption, as experimentally validated in subsequent sections.

In the proposed method, both the spectral encryption and pixel scrambling stages alter only the positional arrangement of the data without modifying the original pixel values. Meanwhile, the DN value diffusion process based on DNA rules is a reversible bit-level operation that incurs no loss of information. Therefore, the proposed hyperspectral image encryption algorithm supports lossless decryption.

The 2D-CSCM is a deterministic function, yet its hyperchaotic sensitivity means that even minor floating-point discrepancies across different IEEE 754-compliant systems could, in theory, cause trajectory divergence after many iterations. A pivotal design choice in our algorithm inherently mitigates this risk: the entire process—from key generation through all chaotic sequence iterations required for encryption—is executed as a single, continuous computational workflow on the encryption machine. Consequently, only the final ciphertext and the initial key (*kx*, *ky*, *ku*) are shared. The decryption process does not resume from an intermediate state; it independently regenerates the entire chaotic sequence from the shared initial key. This approach ensures that any platform-specific numerical behavior originates from the same starting point, preventing the accumulation of divergent errors that could occur if intermediate states were transferred.

To empirically validate this design, we conducted a cross-platform test. A ZY1E hyperspectral image was encrypted on a local workstation (Intel i9, Windows 11). The ciphertext and key were then decrypted on Google Colab (cloud environment) and a Linux server (Ubuntu 22.04). The decrypted images on both platforms were bit-for-bit identical to the original (MSE = 0, SSIM = 1.0), confirming the practical robustness of our design for lossless decryption across heterogeneous environments.

## 4. Experimental Results and Performance Evaluation

This section presents the experimental results and performance evaluations of the proposed hyperspectral remote sensing image encryption algorithm. The first subsection demonstrates the effect and quantitative metrics of spectral-domain encryption, evaluating the encryption performance of the spectrally encrypted image P0. Subsections 2 to 9 present the overall encryption effect of the complete algorithm and report various performance metrics commonly used in chaotic image encryption.

Experiments were conducted on four hyperspectral remote sensing images:

ZY1E ASHI Subset: A subset of images acquired by the ASHI sensor onboard the ZY-1-02D satellite. Size: [M, N, K] = [512, 512, 76]. Data Type: 16-bit unsigned integer (DN values).

Botswana: Captured by NASA’s EO-1 satellite. Size: [200, 200, 145]. Data Type: 16-bit unsigned integer. (Source: GIC Website).

PaviaU: Collected by the ROSIS sensor. Size: [610, 340, 103]. Data Type: 16-bit unsigned integer. (Source: GIC Website).

Indian Pines: Acquired by the AVIRIS sensor. Size: [145, 145, 200]. Data Type: 16-bit unsigned integer. (Source: GIC Website).

(Hyperspectral Remote Sensing Scenes—Grupo de Inteligencia Computacional (GIC)).

All experiments were conducted on a computer with the following configuration: Intel Core i9-13900H CPU @ 2.20 GHz, 16 GB RAM, and the Windows 11 operating system. The algorithm was implemented in Python 3.7.

### 4.1. Spectral Domain Information Encryption Capability

#### 4.1.1. Encrypted Spectral Profile Comparison

**Figure 9:**
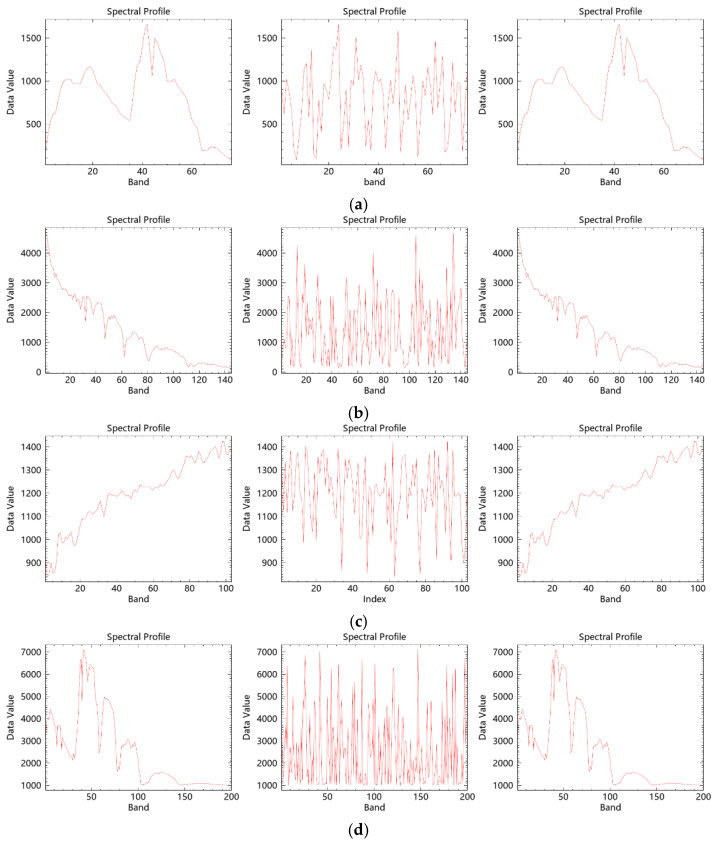
Comparison of original, encrypted, and decrypted spectral profiles of hyperspectral remote sensing images. (**a**) Performance of ZY1E Satellite Hyperspectral Images at (100, 100); (**b**) Performance of Botswana at (100, 100); (**c**) Performance of PaviaU at (100, 100); (**d**) Performance of Indian Pines at (100, 100).

Hyperspectral remote sensing images possess high spectral resolution, enabling the extraction of intensity values across all bands at any pixel to form an approximately continuous spectral curve. To evaluate the effectiveness of spectral encryption, a specific pixel at location (100, 100) was selected, and its spectral profiles were extracted from the original, encrypted, and decrypted image data blocks. Figure 9 displays the spectral curves from the original, encrypted, and decrypted images for four hyperspectral datasets. The encrypted spectral profiles differ significantly from the original ones, effectively concealing the initial spectral characteristics, while the decrypted spectra are fully restored to their original forms, demonstrating that the proposed algorithm achieves efficient encryption and lossless decryption in the spectral domain.

**Figure 10:**
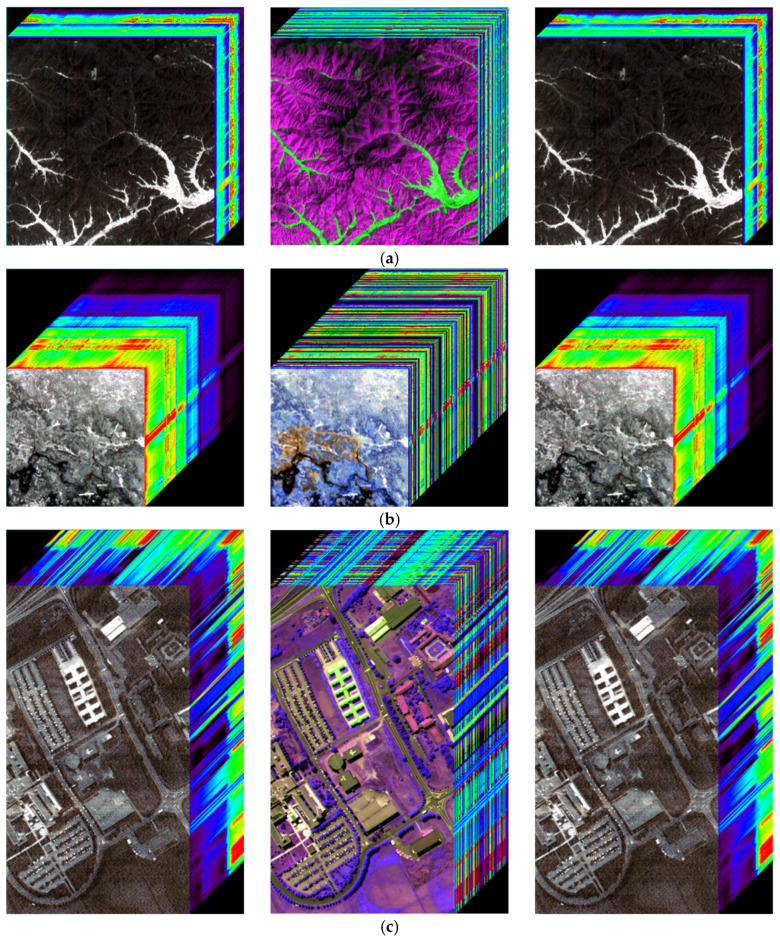

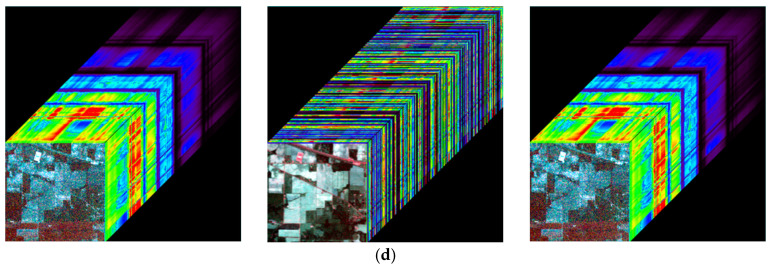
Image cubes before encryption, after spectral encryption, and after decryption. (**a**) Performance of ZY1E Satellite Hyperspectral Images; (**b**) Performance of Botswana; (**c**) Performance of PaviaU; (**d**) Performance of Indian Pines.

Furthermore, the encryption algorithm independently encrypts spectral profiles without altering spatial information. Figure 10 shows the image cubes of the original, spectrally encrypted, and decrypted images. The spectral cross-sections of the encrypted image cubes are substantially altered, whereas the spatial information remains unchanged. The decrypted image cubes revert entirely to their original state. These results confirm that the proposed algorithm securely encrypts spectral information while fully preserving spatial content.

#### 4.1.2. Quantitative Evaluation

To quantitatively evaluate the similarity between the original spectral curves and the encrypted/decrypted ones, this study employs two metrics: the Spectral Angle Mapper (SAM) and the Spectral Information Divergence (SID) [49].

SAM measures the similarity between two spectral vectors by calculating the angle between them. For two spectral curves s and $s^{\prime }$ of length L, the SAM value is computed as:

(17) $$SAM\left(s,s^{\prime }\right)={cos}^{-1}\left(\frac{\langle s,s^{\prime }\rangle }{\left\|s\right\|\left\|s^{\prime }\right\|}\right)$$

where $\langle s,s^{\prime }\rangle =\sum_{l=1}^{L}s_{l}{s_{l}}^{\prime }$, $\left\|s\right\|=\sqrt{\sum_{l=1}^{L}{{(s}_{l})}^{2}}$ and $\left\|s^{\prime }\right\|=\sqrt{\sum_{l=1}^{L}{{{(s}_{l}}^{\prime })}^{2}}$.

SID treats spectral curves as random variables and measures their similarity through the divergence of their probability distributions. Let $x={\left(x_{1},x_{2},\ldots ,x_{L}\right)}^{T}$ and $y={\left(y_{1},y_{2},\ldots ,y_{L}\right)}^{T}$ be two spectral pixel vectors. Their probability distributions are obtained by normalization: pl=xl∑l=1Lxl, ql=yl∑l=1Lyl.Then the SID is defined as:

(18) $$SID\left(x,y\right)=D\left(x∥y\right)+D\left(y∥x\right)$$

where:

(19) $$\left\{\begin{array}{c} D(x||y)=\sum_{l=1}^{L}p_{l}\log\left(\frac{p_{l}}{q_{l}}\right)\\ D(y||x)=\sum_{l=1}^{L}q_{l}\log\left(\frac{q_{l}}{p_{l}}\right) \end{array}\right.$$

Smaller SAM and SID values indicate higher similarity. As shown in Table 2, the SAM and SID values increase significantly after encryption, but both drop to zero after decryption, demonstrating that the encryption effectively disrupts the original spectral features while allowing perfect recovery, thus confirming the algorithm’s effectiveness and reversibility in spectral encryption.

### 4.2. Visual Effects

**Figure 11:**
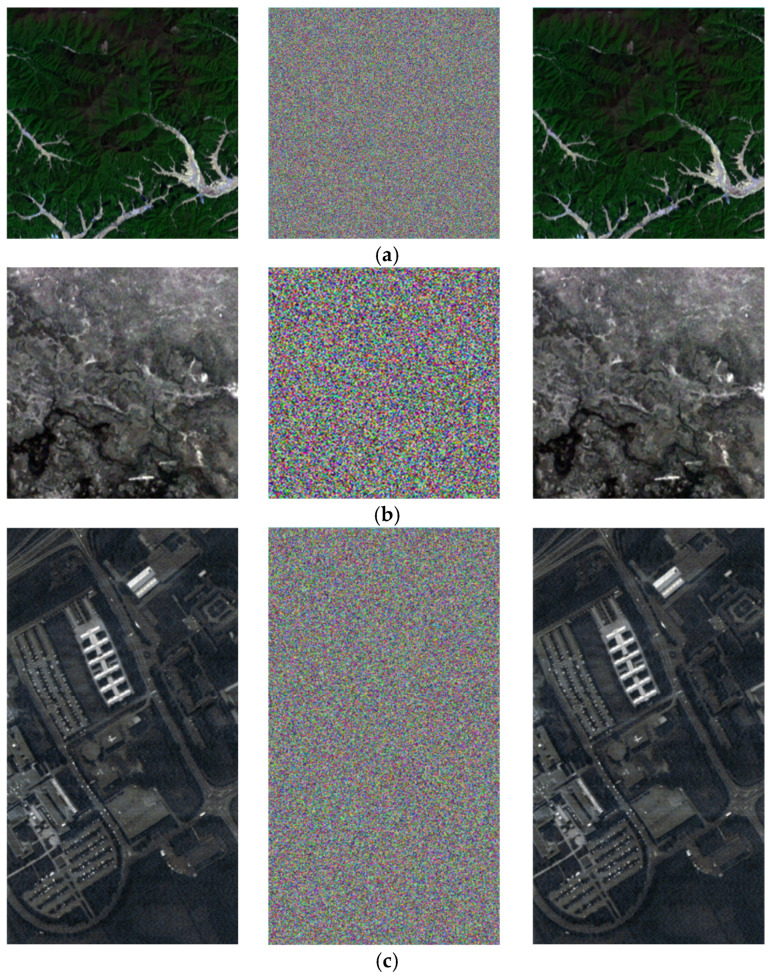

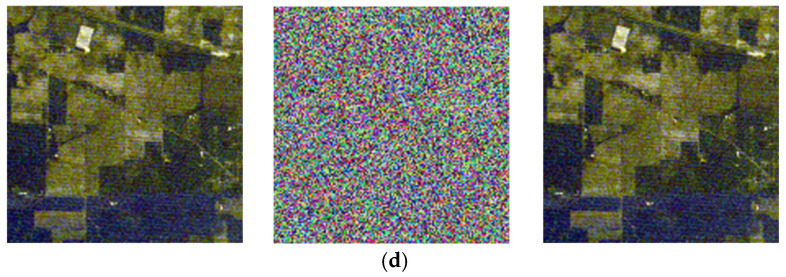
Visual comparison of hyperspectral remote sensing images before encryption, after encryption, and after decryption. (**a**) Performance of ZY1E Satellite Hyperspectral Images (Band selection: R = 32, G = 21, B = 11); (**b**) Performance of Botswana (Band selection: R = 3, G = 2, B = 1); (**c**) Performance of PaviaU (Band selection: R = 3, G = 2, B = 1); (**d**) Performance of Indian Pines (Band selection: R = 3, G = 2, B = 1).

**Figure 12:**
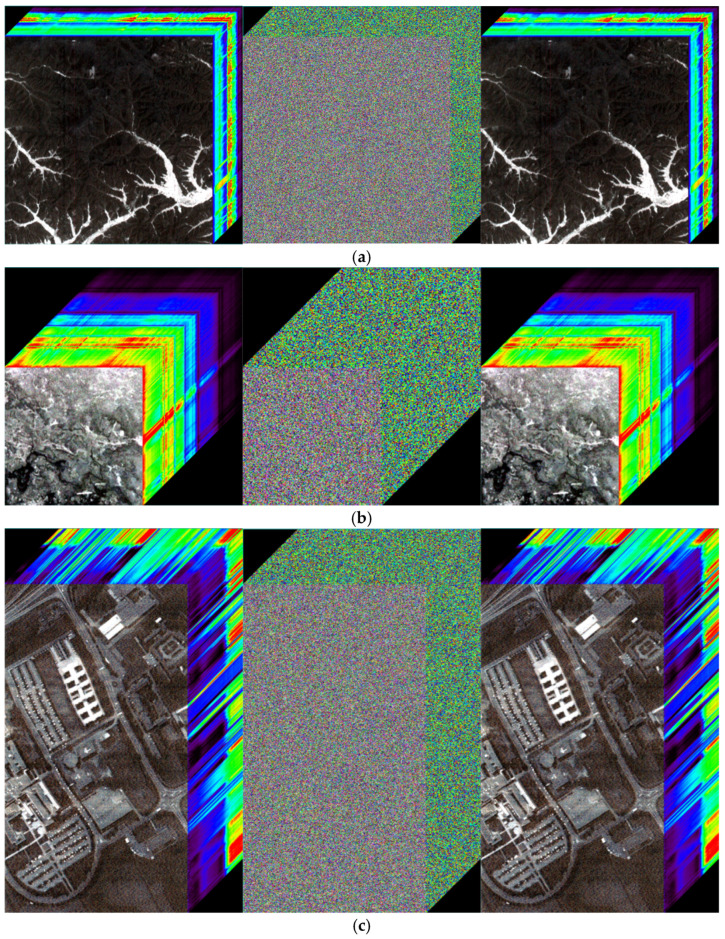

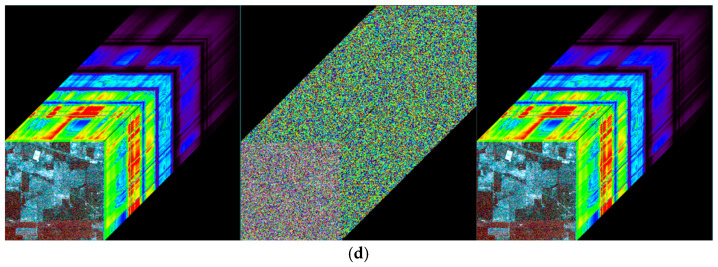
Image cubes before encryption, after encryption, and after decryption. (**a**) Performance of ZY1E Satellite Hyperspectral Images; (**b**) Performance of Botswana; (**c**) Performance of PaviaU; (**d**) Performance of Indian Pines.

The visual effects of the proposed encryption algorithm are illustrated in Figure 11 and Figure 12. Figure 11 compares the original, encrypted, and decrypted images in three selected spectral bands, while Figure 12 shows the corresponding image cubes before encryption, after encryption, and after decryption. It can be observed that the encrypted images and image cubes completely conceal the visual information of the originals, exhibiting a noise-like random distribution. Meanwhile, the decrypted images are visually identical to the original ones without any distortion or loss of information. These results demonstrate that the proposed encryption algorithm effectively protects the visual content of hyperspectral remote sensing images while achieving lossless decryption.

### 4.3. Key Space Analysis

In encryption systems, a key space larger than 2^100^ is generally considered sufficient to resist brute-force attacks, meeting common security standards [50]. In the proposed algorithm, the effective secret key comprises the three floating-point numbers ku, kx and ky. In digital computers, the precision of these floating-point numbers is finite. According to the IEEE 754 double-precision standard, each double-precision floating-point number has a precision of about $10^{-15}$, effectively providing approximately $2^{52}$ distinct representable values within a given range.

Therefore, the conservative lower bound for the key space can be calculated as: ($2^{52}$) × ($2^{52}$) × ($2^{52}$) = $2^{156}$. This key space of $2^{156}$ not only far exceeds the common security threshold of $2^{100}$ but is also fully compliant with the key space sizes achieved in state-of-the-art chaotic image encryption schemes. This demonstrates that the proposed algorithm possesses a sufficiently large key space to effectively resist brute-force attacks.

### 4.4. Key Sensitivity Analysis

**Figure 13:**
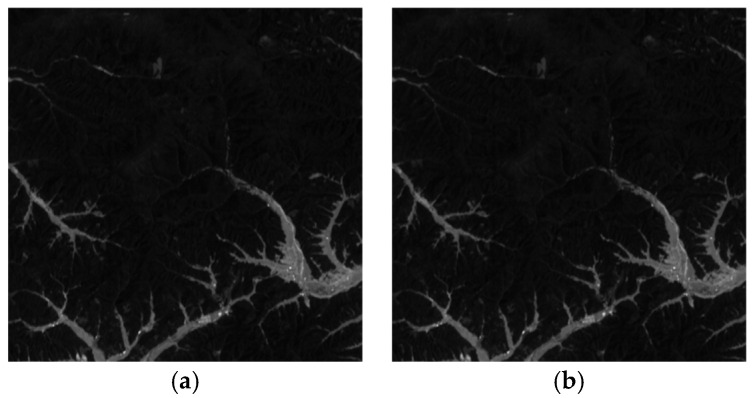
Original Band 7 image of ZY1E dataset and decryption result with correct key. (**a**) Original image; (**b**) Image decrypted with the correct key.

**Figure 14:**
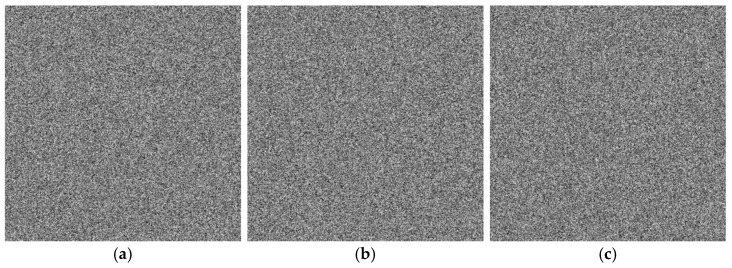
Decrypted images with perturbed keys: ku, kx, and ky. (**a**) Image decrypted using $ku+10^{-15}$; (**b**) Image decrypted using $kx+10^{-15}$; (**c**) Image decrypted using $ky+10^{-15}$.

**Figure 15:**
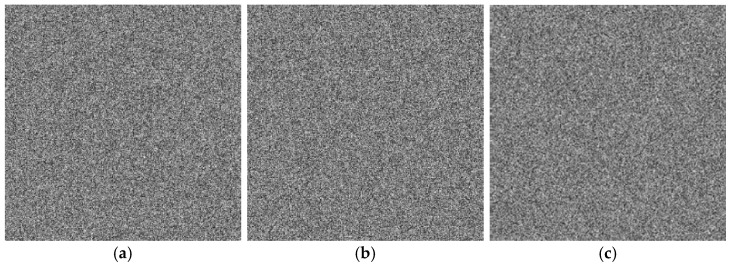
DN value differences between original and decrypted images using perturbed keys. (**a**) ku; (**b**) kx; (**c**) ky.

**Figure 16:**
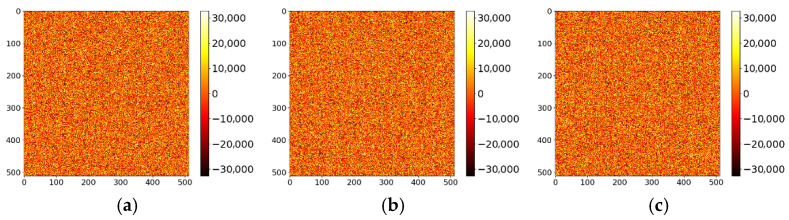
Heatmaps of DN value differences for decryptions with perturbed keys. (**a**) ku; (**b**) kx; (**c**) ky.

Key sensitivity refers to the property that even a minor modification to the key should make it impossible to decrypt the ciphertext correctly. A secure image encryption algorithm must exhibit high key sensitivity [51]. This section evaluates the key sensitivity of the proposed algorithm. A slight perturbation Δ = 10^−15^ was introduced to the original key components ku, kx, and ky individually to generate incorrect keys. Figure 13a shows the original Band 7 image of the ZY1E satellite hyperspectral dataset, and Figure 13b displays the image decrypted with the correct key. Figure 14 presents the decryption results using $ku+10^{-15}$, $kx+10^{-15}$, and $ky+10^{-15}$, respectively. It is evident that decryption with slightly altered keys fails to recover any recognizable information. Figure 15 illustrates the DN value differences between the incorrectly decrypted images and the original image, while Figure 16 provides heatmaps of these differences. The differences are significant and irregular, confirming the complete decryption failure. These results demonstrate the high key sensitivity of the proposed encryption algorithm, highlighting its security against key-based attacks.

### 4.5. Statistical Analysis

In cryptanalysis of image encryption algorithms, a common approach is to exploit statistical features of the ciphertext image. These characteristics can also be analyzed through their histograms, which reflect the distribution patterns of pixel values. A flatter histogram indicates weaker correlation between pixel values and their frequencies, making it more difficult to decode the original image information [52]. As the encryption target in this paper is hyperspectral remote sensing images whose pixel values are Digital Number (DN) values, histogram tests are performed on the DN values.

**Figure 17:**
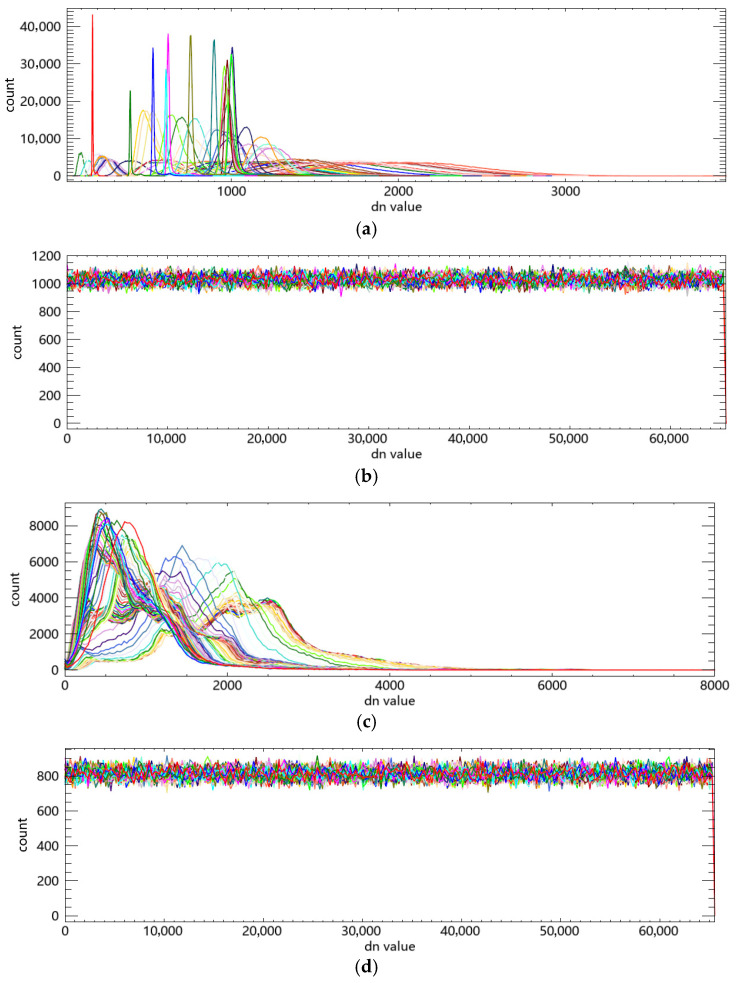
Comparison of DN value histograms before and after encryption. (**a**) DN value histogram of ZY1E hyperspectral image before encryption; (**b**) DN value histogram of ZY1E hyperspectral image after encryption; (**c**) DN value histogram of PaviaU image before encryption; (**d**) DN value histogram of PaviaU image after encryption.

The histogram test results of the proposed encryption algorithm are shown in Figure 17. Figure 17a,b show the DN value histograms of the ZY1E satellite hyperspectral image before and after encryption, respectively; Figure 17c,d show those of the PaviaU image before and after encryption. The original images exhibit distinct statistical characteristics in their DN value distributions. After encryption, the histograms of the encrypted images become uniform across the entire DN value range. This indicates that the proposed encryption algorithm successfully conceals the statistical features of the images, making the encrypted images resistant to statistical analysis and histogram-based attacks.

### 4.6. Correlation Analysis

Correlation analysis is another statistical method used to examine the relationships between adjacent pixels in an image, which could be exploited by attackers [53]. Therefore, an image encryption algorithm should enhance data security by disrupting the correlation between neighboring pixels. To evaluate the security of the proposed algorithm, we randomly selected 10,000 pixels from the image and calculated the correlation coefficients between adjacent pixels in the horizontal (H), vertical (V), and diagonal (D) directions. The correlation coefficient is computed using the following formula:

(20) $$\left\{\begin{array}{c} r_{xy}=\frac{cov\left(x,y\right)}{\sqrt{D\left(X\right)}\sqrt{D\left(Y\right)}}\\ cov\left(x,y\right)=\frac{1}{NP}\sum_{I=1}^{N}\left(x_{i}-E\left(x\right)\right)\left(y_{i}-E\left(y\right)\right)\\ D\left(X\right)=\frac{1}{NP}\sum_{i=1}^{N}{\left(x_{i}-E\left(x\right)\right)}^{2}\\ E\left(x\right)=\frac{1}{NP}\sum_{i=1}^{N}x_{i} \end{array}\right.$$

where x and y represent the values of two adjacent pixels, NP is the number of selected pixels, $cov\left(x,y\right)$ denotes the covariance, $D\left(X\right)$ is the variance, and $E\left(x\right)$ is the mean value. A value of $r_{xy}$ close to 1 indicates high correlation, while a value near 0 suggests low correlation.

**Figure 18:**
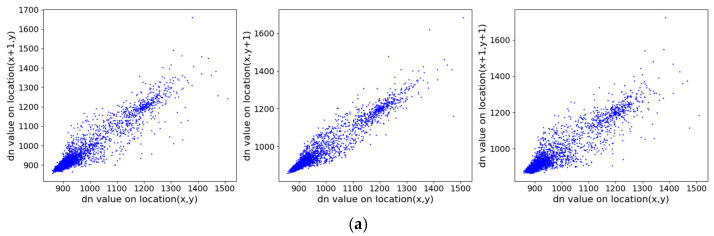

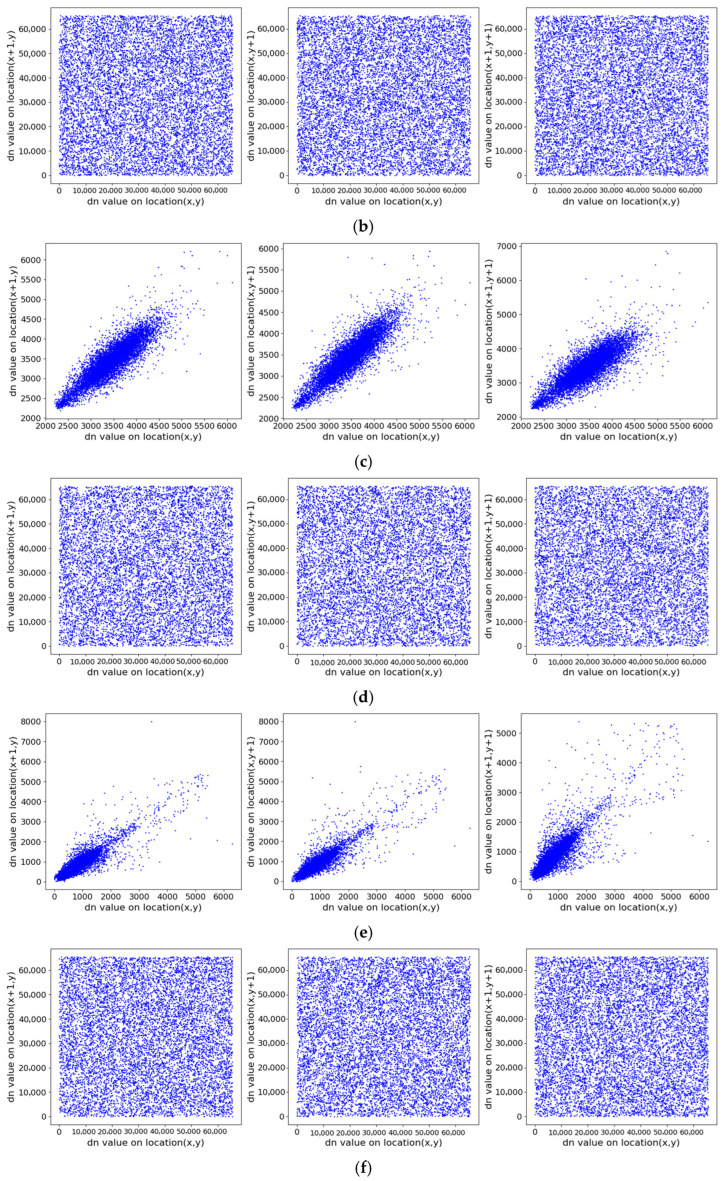

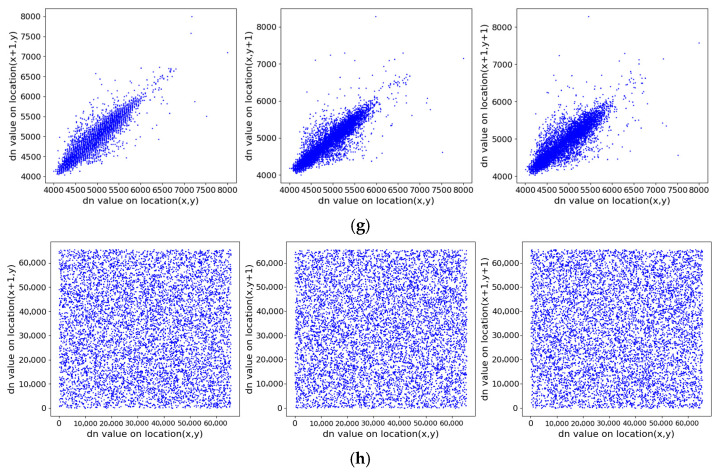
Correlation distributions of adjacent pixels in original and encrypted images on three directions (horizontal, vertical, diagonal). (**a**) ZY1E before encryption; (**b**) ZY1E after encryption; (**c**) Botswana before encryption; (**d**) Botswana after encryption; (**e**) PaviaU before encryption; (**f**) PaviaU after encryption; (**g**) Indian Pines before encryption; (**h**) Indian Pines after encryption.

Figure 18 illustrates the correlation distributions of the original and encrypted images in three directions. The pixel points of the original image are clustered near the diagonal, indicating high correlation, whereas those of the encrypted image are uniformly scattered across the plane, showing significantly reduced correlation. Table 3 provides the specific correlation coefficients: before encryption, the coefficients in all directions are close to 1; after encryption, they are all near 0. These results demonstrate that the proposed encryption algorithm effectively disrupts the correlation between adjacent DN values, thereby resisting statistical attacks based on pixel relationships and enhancing overall security.

### 4.7. Information Entropy

The degree of randomness of an information source can be measured and quantified using information entropy [54]. According to Shannon’s principle, the entropy H(m) of a source mm is calculated as:

(21) $$H\left(m\right)=\sum_{i=1}^{L}p(x_{i})log\frac{1}{p\left(x_{i}\right)}$$

where L is the number of possible intensity levels, and $p(x_{i})$ is the probability of a particular intensity value $x_{i}$ appearing in the image. For a grayscale image with L=256, the ideal entropy is 8. Since the encrypted objects in this paper are hyperspectral remote sensing images with a bit depth of 16 bits, L=65536, and the ideal entropy value is 16. A ciphertext entropy close to the ideal value indicates higher randomness and better encryption performance.

Table 4 lists the information entropy values of the original and encrypted images, alongside the ideal value for 16-bit data. The entropy of the original images is considerably lower than 16, confirming that their DN value distributions are far from random, a common characteristic of real-world hyperspectral scenes. After encryption, the entropy increases significantly and approaches the ideal value, demonstrating that the encrypted images exhibit high randomness. It is noted that the encrypted entropy for the ‘Botswana’ and ‘Indian Pines’ datasets is slightly lower than the ideal maximum. This is not a limitation of the encryption algorithm but stems from the inherent data characteristics of the original images, which contain large homogeneous areas (e.g., shadows, uniform vegetation) that do not utilize the full 16-bit dynamic range. The algorithm successfully maximizes the randomness to a level very close to the theoretical ceiling permitted by the source data. These results confirm the effectiveness of the proposed algorithm in enhancing randomness and resisting entropy-based attacks.

### 4.8. Resistance to Cropping Attacks

Robustness is a crucial metric for evaluating the ability of an image encryption algorithm to withstand disturbances or attacks, with resistance to cropping attacks being particularly important. During data transmission, images may suffer from partial information loss due to channel noise or malicious interventions [55]. To assess the algorithm’s resilience against such disturbances, this experiment simulates cropping attacks by intentionally removing portions of the encrypted image and examining whether the original image can still be recovered recognizably.

**Figure 19:**
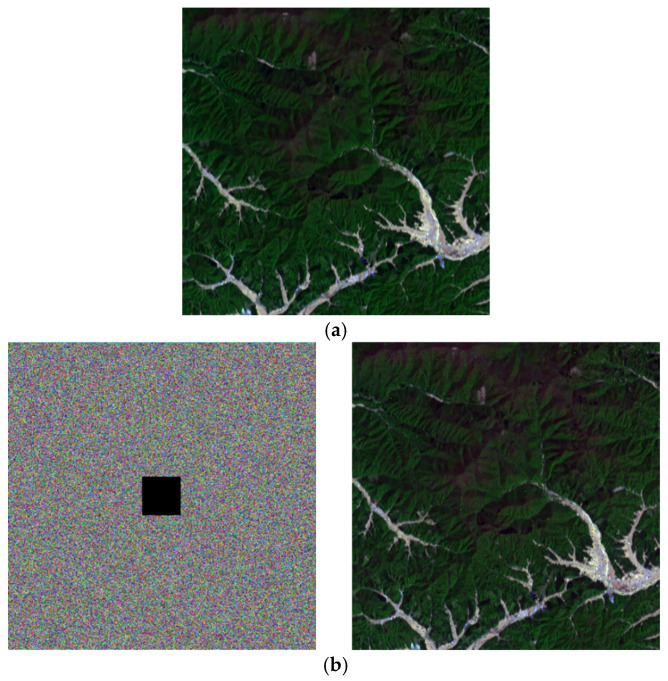

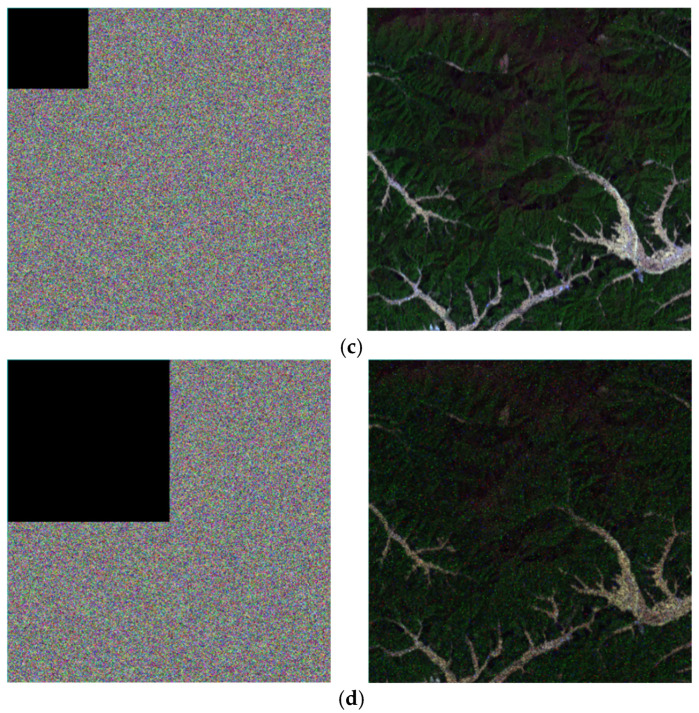
Experimental results of resistance to cropping attacks. (**a**) Original image (Band selection: R = 32, G = 21, B = 11); (**b**) Cropped 1/64 region and decrypted image; (**c**) Cropped 1/16 region and decrypted image; (**d**) Cropped 1/4 region and decrypted image.

Figure 19a shows the original ZY1E satellite hyperspectral image in a band combination (R:32, G:21, B:11); Figure 19b–d present the decryption results after cropping 1/64, 1/16, and 1/4 of the encrypted image, respectively. The results demonstrate that despite varying degrees of data loss, the algorithm successfully decrypts the images, with primary geographic information preserved in the reconstructed images. These findings indicate that the proposed encryption algorithm exhibits strong robustness against cropping attacks, making it suitable for practical scenarios where partial data corruption may occur during transmission.

### 4.9. Resistance to Differential Attacks

Differential attacks are a type of chosen-plaintext attack. The core idea is to analyze the impact of differences in specially chosen plaintext pairs on the resulting ciphertext differences, thereby inferring the most probable key [56]. An algorithm resistant to differential attacks should ensure that a minor change in the plaintext leads to significant alterations in the ciphertext. The Number of Pixels Change Rate (NPCR) and the Unified Average Changing Intensity (UACI) are two primary metrics for evaluating this property [57]. The corresponding formulas are as follows:

(22) $$\left\{\begin{array}{c} NPCR=\frac{1}{M\times N}\sum_{i=1}^{W}\sum_{j=1}^{H}D\left(i,j\right)\times 100\%\\ UACI=\frac{1}{M\times N}\left\{\sum_{i=1}^{W}\sum_{j=1}^{H}\frac{\left|C_{1}\left(i,j\right)-C_{1}\left(i,j\right)\right|}{255}\right\}\times 100\% \end{array}\right.$$

where M and N are the number of rows and columns of the image, $C_{1}$ and $C_{2}$ are two ciphertext images whose original plaintexts differ by only one pixel value, D(i,j) is an indicator function (1 if $C_{1}(i,j)\neq C_{2}(i,j)$, else 0), and L is the pixel value range (for 16-bit DN values, L=65535).

The acceptable intervals for NPCR and UACI at a significance level α=0.05 are given by:

(23) $$\left\{\begin{array}{c} NPCR>\frac{L-\phi^{-1}\left(\alpha \right)\sqrt{\frac{L}{M\times N}}}{L+1}\\ \frac{L+2}{3\times L+3}-\phi^{-1}\left(\frac{\alpha }{2}\right)\sqrt{\frac{\left(L+2\right)\times \left(L^{2}+2\times L+3\right)}{18\times {\left(L+1\right)}^{2}\times L\times M\times N}}\leq UACI\\ \leq \frac{L+2}{3\times L+3}+\phi^{-1}\left(\frac{\alpha }{2}\right)\sqrt{\frac{\left(L+2\right)\times \left(L^{2}+2\times L+3\right)}{18\times {\left(L+1\right)}^{2}\times L\times M\times N}} \end{array}\right.$$

where $\phi^{-1}\left(\alpha \right)=1.645$ and $\phi^{-1}\left(\frac{\alpha }{2}\right)=1.96$.

Due to the different value range of DN values in hyperspectral images (L=65,535) compared to ordinary grayscale images, the confidence intervals differ accordingly. Table 5 and Table 6 list the NPCR and UACI values of the test images, along with their confidence intervals and test results. The results show that all NPCR and UACI values fall within the theoretically acceptable ranges, indicating that the proposed algorithm effectively resists differential attacks.

### 4.10. Computational Efficiency Analysis

While the primary focus of this work is on achieving high security for hyperspectral images, we acknowledge the importance of computational efficiency given the large data volume. Experimental results showed that the average encryption times for the ZY1E, Botswana, PaviaU, and Indian Pines datasets were approximately 85.3 s, 23.6 s, 89.1 s, and 19.4 s, respectively.

The results indicate that the encryption time is approximately proportional to the total number of pixels (M×N×K). The proposed algorithm provides a feasible solution for the secure offline storage and transmission of hyperspectral data. Future work will focus on optimizing the algorithm’s speed through parallel computing (e.g., GPU acceleration) to meet real-time requirements.

## 5. Discussion

Although the algorithm performs excellently in security and functional implementation, it still has certain limitations. The computational complexity is relatively high, which may restrict its use in large-scale scenarios with stringent real-time requirements; the current algorithm focuses only on full-image encryption without considering the sensitivity weight of different content within hyperspectral images; furthermore, hyperspectral data possess more underlying features—such as floating-point DN values and internal correlations within the image cube—that could be leveraged to further enhance encryption performance.

Future research efforts could be directed toward the following areas:

To improve computational efficiency, parallel computing and hardware acceleration strategies could be explored. By utilizing parallel computing architectures such as GPUs to parallelize spectral scrambling and DNA diffusion operations, the algorithm’s throughput could be significantly enhanced, meeting the demands of real-time encryption for large-scale hyperspectral data. The proposed method shows notable potential for parallelization, enabling a substantial increase in encryption efficiency while maintaining security through computational architecture optimization.

To enhance algorithm adaptability, novel encryption mechanisms integrating deep learning should be thoroughly investigated. Employing convolutional neural networks to automatically extract image texture features and adaptively adjust encryption strength can improve the algorithm’s intelligence and generalization capability without compromising security.

To broaden the application scope, the algorithm’s use in encrypting multidimensional remote sensing data should be actively explored. Extending the encryption framework to multispectral images, remote sensing video streams, and other geospatial data types will address key technical challenges in integrated security protection for multi-source remote sensing data and provide technical support for building an integrated space-air-ground secure transmission system.

To promote practical deployment, the implementation and optimization of the algorithm on dedicated hardware platforms should be emphasized. Designing hard-ware accelerators for the encryption algorithm using FPGAs or ASICs can meet the application requirements of resource-constrained scenarios such as onboard real-time processing and edge computing, facilitating the translation of research achievements into practical applications.

In summary, the proposed encryption algorithm demonstrates strong performance in protecting hyperspectral remote sensing images, yet there remains room for further optimization. Future research may focus on developing adaptive encryption mechanisms, exploring chaotic map parameters that dynamically evolve with image statistics, and extending the encryption framework to a wider range of geospatial data types. These research directions not only hold significant theoretical value but are also crucial for advancing the practical application of remote sensing data security protection.

## 6. Conclusions

This paper proposes a hyperspectral remote sensing image encryption algorithm based on a 2D-CSCM hyperchaotic map, effectively addressing the encryption challenges posed by the multidimensionality, large volume, and spectral characteristics of hyperspectral data. Experimental results demonstrate that the algorithm achieves efficient encryption of the spectral domain while fully preserving spatial information integrity. It also incorporates an adapted encryption mechanism tailored to the unique data structure and high bit-depth DN values of hyperspectral images, accomplishing comprehensive encryption of hyperspectral remote sensing images.

First, the 2D-CSCM hyperchaotic map was designed and validated through multiple analytical methods, including Lyapunov exponents, sample entropy, and NIST tests, confirming its excellent randomness and complex dynamic behavior, making it highly suitable for high-security image encryption applications. For key generation, the algorithm employs the SHA-512 hash function to derive keys from the original image, ensuring strong correlation between the keys and plaintext. During encryption, the algorithm first performs spectral dimension scrambling to conceal spectral curve features while maintaining the readability of spatial textures in individual bands. It then applies spatial pixel scrambling and DNA encoding-based bit-level diffusion to protect overall spatial texture information, thereby achieving encryption of the entire hyperspectral image. Experiments on real remote sensing images verify the security and effectiveness of the algorithm. The comprehensive experimental results indicate that the algorithm offers a large key space (2^512), significantly increased information entropy close to the ideal value after encryption, very low adjacent pixel correlation, and strong resistance against statistical analysis, cropping attacks, and differential attacks, fully demonstrating its superior security performance and practical value.

In terms of application value, the proposed 2D-CSCM chaotic map exhibits significantly better chaotic performance than existing maps, making it applicable not only to hyperspectral image encryption but also to other image encryption algorithms. By addressing the characteristics of hyperspectral remote sensing images and achieving both spectral and full-image encryption, the algorithm holds considerable application potential in the storage, transmission, and utilization of hyperspectral images.

## Figures and Tables

**Table 1:** NIST SP 800-22 Test Results.

No.	Test Name	*p*-Value	Result
1	Frequency	0.072347	PASS
2	BlockFrequency	0.345215	PASS
3	CumulativeSums	0.345204	PASS
4	Runs	0.712325	PASS
5	LongestRun	0.554398	PASS
6	Rank	0.387691	PASS
7	FFT	0.289123	PASS
8	NonOverlappingTemplate	0.913414	PASS
9	OverlappingTemplate	0.309877	PASS
10	Universal	0.713309	PASS
11	ApproximateEntropy	0.035801	PASS
12	RandomExcursions	0.739918	PASS
13	RandomExcursionsVariant	0.823451	PASS
14	Serial	0.150485	PASS
15	LinearComplexity	0.076882	PASS

**Table 2:** SAM and SID Quantitative Results.

Sample	SAM	SID
ZY1E Encrypted Spectrum	36.5384	41,096.0198
ZY1E Decrypted Spectrum	0.0	0.0
Botswana Encrypted Spectrum	54.1263	307,314.3358
Botswana Decrypted Spectrum	0.0	0.0
PaviaU Encrypted Spectrum	54.2936	137,898.1950
PaviaU Decrypted Spectrum	0.0	0.0
Indian Pines Encrypted Spectrum	45.7839	397,664.9359
Indian Pines Decrypted Spectrum	0.0	0.0

**Table 3:** Correlation coefficients of image adjacent pixels.

Image	Horizontal	Vertical	Diagonal
ZY1E Before Encryption	0.9486	0.9716	0.9360
ZY1E After Encryption	−0.0030	−0.0075	−0.0007
Botswana Before Encryption	0.8728	0.8769	0.8247
Botswana After Encryption	0.0029	−0.0031	0.0019
PaviaU Before Encryption	0.9052	0.9012	0.8500
PaviaU After Encryption	0.0013	−0.0007	−0.0020
Indian Pines Before Encryption	0.9253	0.9088	0.8728
Indian Pines After Encryption	−0.0019	−0.0021	−0.0001

**Table 4:** Information Entropy Comparison.

Image	Before Encryption	After Encryption	Ideal Value
ZY1E Hyperspectral Image	6.8716	15.8107	16.0000
Botswana	7.5675	14.7537	16.0000
PaviaU	10.6903	15.7525	16.0000
Indian Pines	8.0773	14.0647	16.0000

**Table 5:** NPCR Test Results for Differential Attack Analysis.

Image	L Value	NPCR	NPCR Standard Range	Result
ZY1E Hyperspectral Image	65,535	99.9984%	>99.9972%	PASS
Botswana	65,535	100.0%	>99.9952%	PASS
PaviaU	65,535	99.9985%	>99.9970%	PASS
Indian Pines	65,535	100.0%	>99.9940%	PASS

**Table 6:** UACI Test Results for Differential Attack Analysis.

Image	L Value	UACI	UACI Standard Range	Result
ZY1E Hyperspectral Image	65,535	33.2990%	33.2436%~33.4240%	PASS
Botswana	65,535	33.2454%	33.1028%~33.5648%	PASS
PaviaU	65,535	33.2753%	33.2323%~33.4352%	PASS
Indian Pines	65,535	33.5690%	33.0152%~33.6524%	PASS

## Data Availability

The hyperspectral remote sensing image data used in this study were partly obtained from the public dataset available at: https://www.ehu.eus/ccwintco/index.php/Hyperspectral_Remote_Sensing_Scenes, accessed on 3 October 2025 (including scenes such as Pavia Centre and University). The remaining portion of the data is subject to confidentiality agreements and institutional policies, and thus is not publicly available or shared in any form as part of this study.

## Appendix A

To ensure reproducibility and a fair comparison in Section 2.2, the parameters for the compared chaotic maps were set as follows, based on their original publications or standard chaotic regions:

Henon Map: a = 1.4, b = 0.3

2D-SLMM [18]: a = 1, b = 3

2D-LSMCL [19]: α = 0.75, β = 3

2D-CLSS [21]: *p* = 2.5

All maps were evaluated with initial conditions (0.4, 0.6) unless otherwise specified for a particular test.

**Table A1:** Eight kinds of DNA coding rules.

Rules	0	1	2	3	4	5	6	7
A	00	00	01	01	10	10	11	11
T	11	11	10	10	01	01	00	00
G	01	10	00	11	00	11	01	10
C	10	01	11	00	11	00	10	01
